# Triangulating evidence from observational and Mendelian randomization studies of ketone bodies for cognitive performance

**DOI:** 10.1186/s12916-023-03047-7

**Published:** 2023-09-04

**Authors:** Wichanon Sae-jie, Suangsuda Supasai, Mika Kivimaki, Jackie F. Price, Andrew Wong, Meena Kumari, Jorgen Engmann, Tina Shah, Amand F. Schmidt, Tom R. Gaunt, Aroon Hingorani, Pimphen Charoen, on behalf of the UCLEB Consortium

**Affiliations:** 1 Department of Mathematics, Faculty of Science, Mahidol University, Bangkok, 10400 Thailand; 2 Department of Molecular Tropical Medicine and Genetics, Faculty of Tropical Medicine, Mahidol University, Bangkok, 10400 Thailand; 3 UCL Brain Sciences, University College London, 149 Tottenham Court Road, London, W1T 7NF UK; 4 Usher Institute, University of Edinburgh, Edinburgh, EH8 9AG UK; 5 MRC Unit Lifelong Health and Ageing at UCL, London, UK; 6 Institute of Cardiovascular Science, Faculty of Population Health, University College London, London, WC1E 6BT UK; 7 Institute for Social and Economic Research, University of Essex, Colchester, UK; 8 UCL British Heart Foundation Research Accelerator, Department of Cardiology, Division Heart and Lungs, University College London, London, UK; 9 MRC Integrative Epidemiology Unit, Bristol Medical School, University of Bristol, Bristol, BS8 2BN UK; 10 NIHR Bristol Biomedical Research Centre, University Hospitals Bristol National Health Service Foundation Trust and University of Bristol, Bristol, UK; 11 Department of Tropical Hygiene, Faculty of Tropical Medicine, Mahidol University, Ratchawithi Road, Ratchathewi, Bangkok, 10400 Thailand; 12 Integrative Computational Bioscience (ICBS) Center, Mahidol University, Bangkok, Thailand

**Keywords:** Ketone bodies, Cognitive performance, Alzheimer’s disease, Mendelian randomization

## Abstract

**Background:**

Ketone bodies (KBs) are an alternative energy supply for brain functions when glucose is limited. The most abundant ketone metabolite, 3-β-hydroxybutyrate (BOHBUT), has been suggested to prevent or delay cognitive impairment, but the evidence remains unclear. We triangulated observational and Mendelian randomization (MR) studies to investigate the association and causation between KBs and cognitive function.

**Methods:**

In observational analyses of 5506 participants aged ≥ 45 years from the Whitehall II study, we used multiple linear regression to investigate the associations between categorized KBs and cognitive function scores. Two-sample MR was carried out using summary statistics from an in-house KBs meta-analysis between the University College London-London School of Hygiene and Tropical Medicine-Edinburgh-Bristol (UCLEB) Consortium and Kettunen et al. (*N* = 45,031), and publicly available summary statistics of cognitive performance and Alzheimer’s disease (AD) from the Social Science Genetic Association Consortium (*N* = 257,841), and the International Genomics of Alzheimer’s Project (*N* = 54,162), respectively. Both strong (*P* < 5 × 10^−8^) and suggestive (*P* < 1 × 10^−5^) sets of instrumental variables for BOHBUT were applied. Finally, we performed *cis*-MR on *OXCT1*, a well-known gene for KB catabolism.

**Results:**

BOHBUT was positively associated with general cognitive function (*β* = 0.26, *P* = 9.74 × 10^−3^). In MR analyses, we observed a protective effect of BOHBUT on cognitive performance (inverse variance weighted: *β*_IVW_ = 7.89 × 10^−2^, *P*_IVW_ = 1.03 × 10^−2^; weighted median: *β*_W-Median_ = 8.65 × 10^−2^, *P*_W-Median_ = 9.60 × 10^−3^) and a protective effect on AD (*β*_IVW_ =  − 0.31, odds ratio: OR = 0.74, *P*_IVW_ = 3.06 × 10^−2^). *Cis*-MR showed little evidence of therapeutic modulation of *OXCT1* on cognitive impairment.

**Conclusions:**

Triangulation of evidence suggests that BOHBUT has a beneficial effect on cognitive performance. Our findings raise the hypothesis that increased BOHBUT may improve general cognitive functions, delaying cognitive impairment and reducing the risk of AD.

**Supplementary Information:**

The online version contains supplementary material available at 10.1186/s12916-023-03047-7.

## Background

Apart from glucose, ketone bodies (KBs) can cross the blood–brain barrier and are considered to be alternative energy in maintaining brain functions [1]. 3-β-Hydroxybutyrate (BOHBUT), one of the most abundant KBs, has been reported to enhance cognitive abilities [2]. BOHBUT plays crucial roles in brain energy supply at the low glucose [3], neurotransmitter regulation [4, 5], reduction of oxidative stress [6], and preventing neuronal cells from pathogenic cellular proteins, including β-amyloid (Aβ) and phosphorylated tau [7, 8] that can contribute to neurodegenerative diseases.

Many studies have suggested that dietary BOHBUT supplementation may have a positive effect on cognitive function. In small experimental studies, BOHBUT enhanced cognitive memory in patients with type 2 diabetes [9]. In addition, BOHBUT showed positive outcomes on memory functions in older adults free of dementia [10], patients with mild cognitive impairment [11], and those with mild-to-moderate Alzheimer’s disease (AD) [8, 12]. Small randomized controlled trials (RCTs) also suggested KB treatment in both AD and diabetes patients may provide neuroprotection [9, 11, 12]. In studies on mice, it was shown that BOHBUT promoted hippocampal brain-derived neurotrophic factor (BDNF) expression (4, 5), a protein which is positively associated with neuronal survival, neurotransmitter regulation, synaptic plasticity, and memory formation whereas a decrease in BDNF is linked to increased risk of neurodegenerative diseases [13, 14]. The hypothesized biological pathway connecting KB metabolism and cognitive functions is shown in Additional file 1: Fig. S1. In addition, BOHBUT has been reported to enhance learning and memory in AD transgenic mice via improvement in neuronal mitochondrial energy [15]. It is unclear whether KBs improve cognitive performance or prevent or delay cognitive impairment in humans as studies verifying the results across disparate lines of evidence are missing, a technique known as triangulation.

In this study, we present a triangulation of evidence using observational studies and Mendelian randomization (MR) studies to assess evidence of the causality of BOHBUT on cognitive performance. First, we used an observational study to investigate the associations between BOHBUT and cognitive functions. Second, we performed MR analyses (a method of using measured variation in associated genes to examine the causal effect of a modifiable exposure on disease in observational studies) to assess the causal relationships of BOHBUT with cognitive performance and AD, a disorder characterized by cognitive impairment. In addition to BOHBUT, we also performed parallel analyses on acetoacetate (ACACE), a precursor to BOHBUT. After fatty acids are broken down in the liver, ACACE is produced before converting to BOHBUT [16, 17]. ACACE is known as an unstable compound that becomes rapidly decarboxylated and is produced in a small amount; thus, it is a less reliable measurement for KBs compared to BOHBUT [18].

## Methods

### Observational study

#### Datasets for observational study

**Fig. 1:**
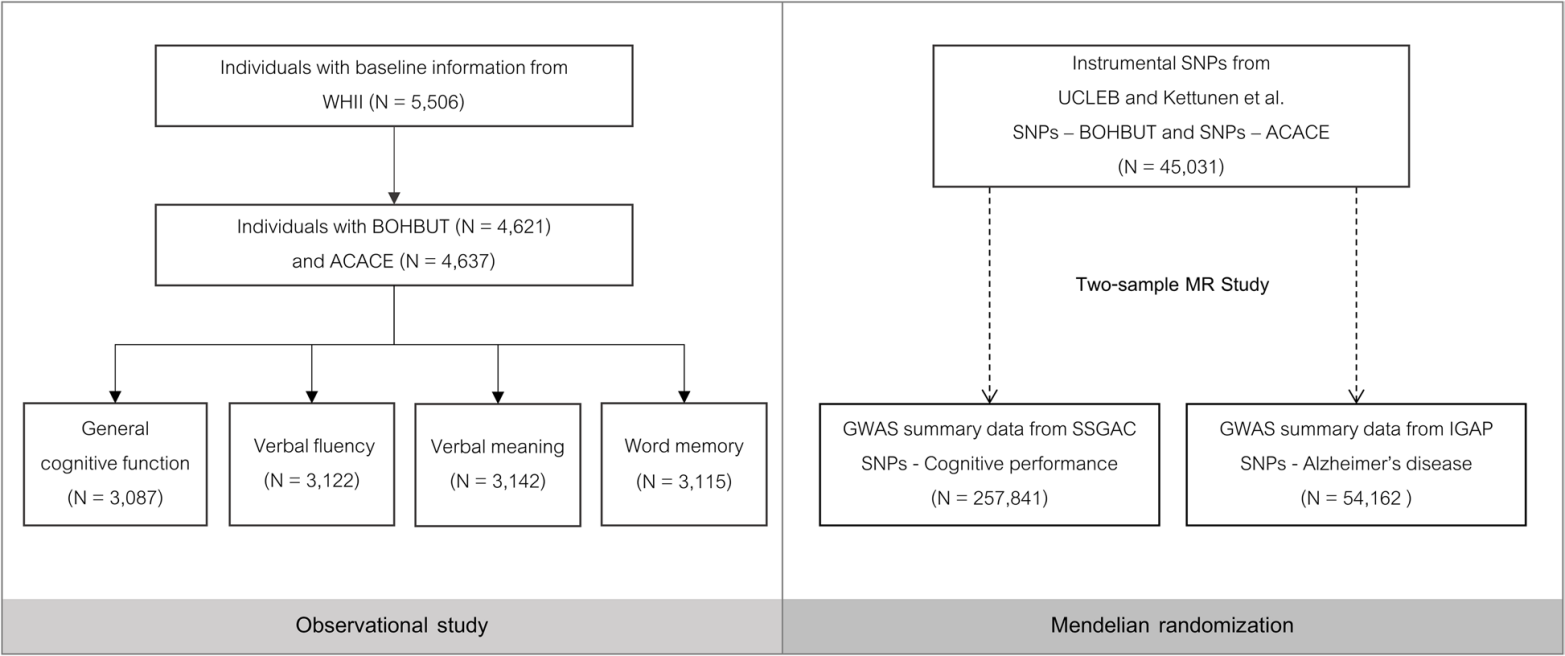
Study setting and dataset. The approach of triangulating evidence from observational and Mendelian randomization studies is used to investigate the associations between ketone bodies and cognitive functions including Alzheimer’s disease. WHII, Whitehall II study; UCLEB, University College-London School-Edinburgh-Bristol Consortium; SSGAC, Social Science Genetic Association Consortium; IGAP, International Genomics of Alzheimer’s Project; BOHBUT, 3-β-hydroxybutyrate; ACACE, acetoacetate; SNPs, single nucleotide polymorphism

To assess the association between KBs and cognitive functions, we used the Whitehall II study (WHII) including 5506 participants from the University College London-London School of Hygiene and Tropical Medicine-Edinburgh-Bristol (UCLEB) Consortium [19] (Fig. 1, Additional file 1: Table S1). Out of the initial 5506 samples, there were 4621 samples with BOHBUT and 4637 sample with ACACE. We exclusively used complete cases in the observational analysis, i.e. individuals with missing covariate data were excluded. Therefore, there were 2214 samples in the BOHBUT observational analysis and 2224 samples in the ACACE observational analysis. Both KB and cognitive functions were measured at baseline (details in Additional file 1: Supplementary Method).

Nuclear magnetic resonance (NMR) spectroscopy was used to measure circulating BOHBUT and ACACE from serum collected after fasting for at least 8 h. Samples that did not meet the quality control criteria during the automated data processing on the NMR platform were excluded. For instance, this applied to those exhibiting degradation and contamination issues. We did not observe any 0 mmol/L of KB values in WHII which could be obtained from the standard procedure in the platform to set metabolite value to zero when its concentration was above the limit of detection but below the limit of quantification due to biological reasons or external compounds interfering with the quantification.

Three types of cognitive function scores were measured using the following psychometric assessments. *Word memory* was assessed by recalling a list of 20 words that participants heard in 2 min (*N* = 3115). *Verbal fluency* was assessed by naming as many animals as possible within 1 min (*N* = 3122). *Verbal meaning* was assessed by answering multiple choice questions for 33 stimulus words ordered by increasing difficulty (*N* = 3142). The details of the protocol are reported in Elovainio et al. [20].

#### Observational analyses

For an outcome, we calculated the “general” cognitive performance score to obtain comparable results to our MR analyses for which we obtained publicly available summary statistics from cognitive performance meta-analysis in the Social Science Genetic Association Consortium (SSGAC) [21]. By avoiding taking more than one cognitive test score from any individual cognitive test, the principal component analysis was applied to cognitive test scores to derive a measure of “general” cognitive performance. The scores on the first unrotated component were used as the general cognitive function [22, 23]. Therefore, using a similar approach, we calculated the scores on the first unrotated component based on verbal meaning, verbal fluency, and word memory in WHII. In addition, the scores from each cognitive test, including verbal meaning, verbal fluency, and word memory, were also used as outcomes in observational analyses.

For exposure, we categorized KBs into two groups, e.g., high vs normal levels, based on the assumption that KBs could have a therapeutic benefit when the concentrations reach a certain level [24]. Due to the unknown cutoff, we varied the thresholds across the KB range. Association analysis between categorized KBs and cognitive function scores was performed using multiple linear regression. Age; sex; diabetes (yes/no); smoking (ever/never); alcohol consumption (heavy/other); adiposity, i.e., waist-to-hip ratio; and occupational position (low/intermediate/high) were used as covariates. KB cutoff was varied under the range that allows the minimum 90% statistical power under the two-sample *t*-test. The unit for the regression coefficient of KB was reported as the standard deviation of cognitive scores per KB group exceeding the cutoff.

Association analyses between continuous KBs and cognitive function scores were also reported. The same set of covariates was applied, and the unit for the regression coefficient of KB was reported as the standard deviation of cognitive scores per 1 mmol/L of KB. As we performed association analyses across 3 different cognitive performances which are not highly correlated (cor_Fluency-Memory_ = 0.32, cor_Fluency-Meaning_ = 0.46, and cor_Memory-Meaning_ = 0.22), a Bonferroni-corrected significance threshold of 0.05/3 = 0.0167 was applied to adjust for multiple testing. A *P*-value < 0.05 was considered a suggestive association, requiring confirmation.

### Mendelian randomization study

#### Datasets for Mendelian randomization

A two-sample MR analysis was applied to incorporate available summary statistics from two genome-wide association studies (GWAS) with non-overlapping samples for exposure and outcome.

#### Exposure data: ketone bodies

We obtained genetic instruments and their summary statistics from our in-house KB meta-analysis between the UCLEB Consortium [19] and Kettunen et al. [25] involving 45,031 participants (Fig. 1, Additional file 1: Supplementary Method). Two sets of single nucleotide polymorphisms (SNPs) as instrumental variables were used: (1) a strong set of instrumental SNPs (*P* < 5 × 10^−8^) and (2) a suggestive set of instrumental SNPs (*P* < 1 × 10^−5^). All instrumental SNPs were ensured independence using *r*^2^ < 0.001 with a clumping window of 10,000 kb. European samples in the 1000 Genomes Project were used as a reference panel for calculating linkage disequilibrium [26].

#### Outcome data: cognitive performance

Summary statistics for SNPs associated with cognitive performance and AD were retrieved from the publicly available data. Cognitive performance involving 257,841 participants was extracted from the SSGAC Consortium [21], and AD involving 54,162 participants was from the International Genomics of Alzheimer’s Project (IGAP) [27]. The details of demographics and GWAS analyses for cognitive performance and AD were provided in Additional file 1: Table S1.

#### Two-sample Mendelian randomization analyses

Firstly, we applied the conventional inverse variance weighted (IVW) method. A random-effect IVW was applied when there was evidence of heterogeneity using Cochran’s *Q* test (*P* < 0.1); otherwise, a fixed-effect IVW was applied [28]. For sensitivity analyses, we applied various methods to assess the robustness of results when MR assumptions are deviated: weighted median method (W-Median), weighted mode-based estimation method (W-Mode), MR-Egger regression, and Mendelian Randomization Pleiotropy RESidual Sum and Outlier (MR-PRESSO). The W-Median method provides valid causal estimates under the assumption that at least half of the contributing information comes from genetic variants that are valid instruments [29]; the W-Mode method allows an unbiased effect estimate when the most common causal effect estimate is consistent with the true causal effect, even if the majority of instruments are invalid [30]; MR-Egger can identify and control for bias due to directional pleiotropy [31]; and MR-PRESSO can detect and correct the effects of outliers that could bias the results [32]. In these analyses, *R*^2^ represents the proportion of variance in liability to KBs explained by SNPs. We reported *F*-statistics to evaluate the strength of instrumental SNPs. The strong set of instruments (*F*-statistic > 10) is desirable; nevertheless, the exclusion of weak instruments may introduce more bias than prevents [33]. Lastly, we conducted *cis*-MR using SNPs with *P* < 1 × 10^−5^ (*r*^2^ < 0.4) and located within 100 kb around the encoding genes and applied IVW and MR-Egger methods accounting for residual correlation [34]. The analyses were performed using the “TwoSampleMR” [26] and the “MendelianRandomization” [35] packages in R.

## Results

### Participant characteristics

Descriptive statistics on the characteristics of the WHII are shown in Additional file 1: Table S2. Participants were European, aged 44 to 70 years, and 72% of them were men. The median of BOHBUT was 0.116 mmol/L (interquartile range: IQR 0.092), and ACACE was 0.044 mmol/L (IQR 0.043). The cognitive function scores were standardized (mean = 0, SD = 1) to allow comparison between tests.

### Observational study

**Fig. 2:**
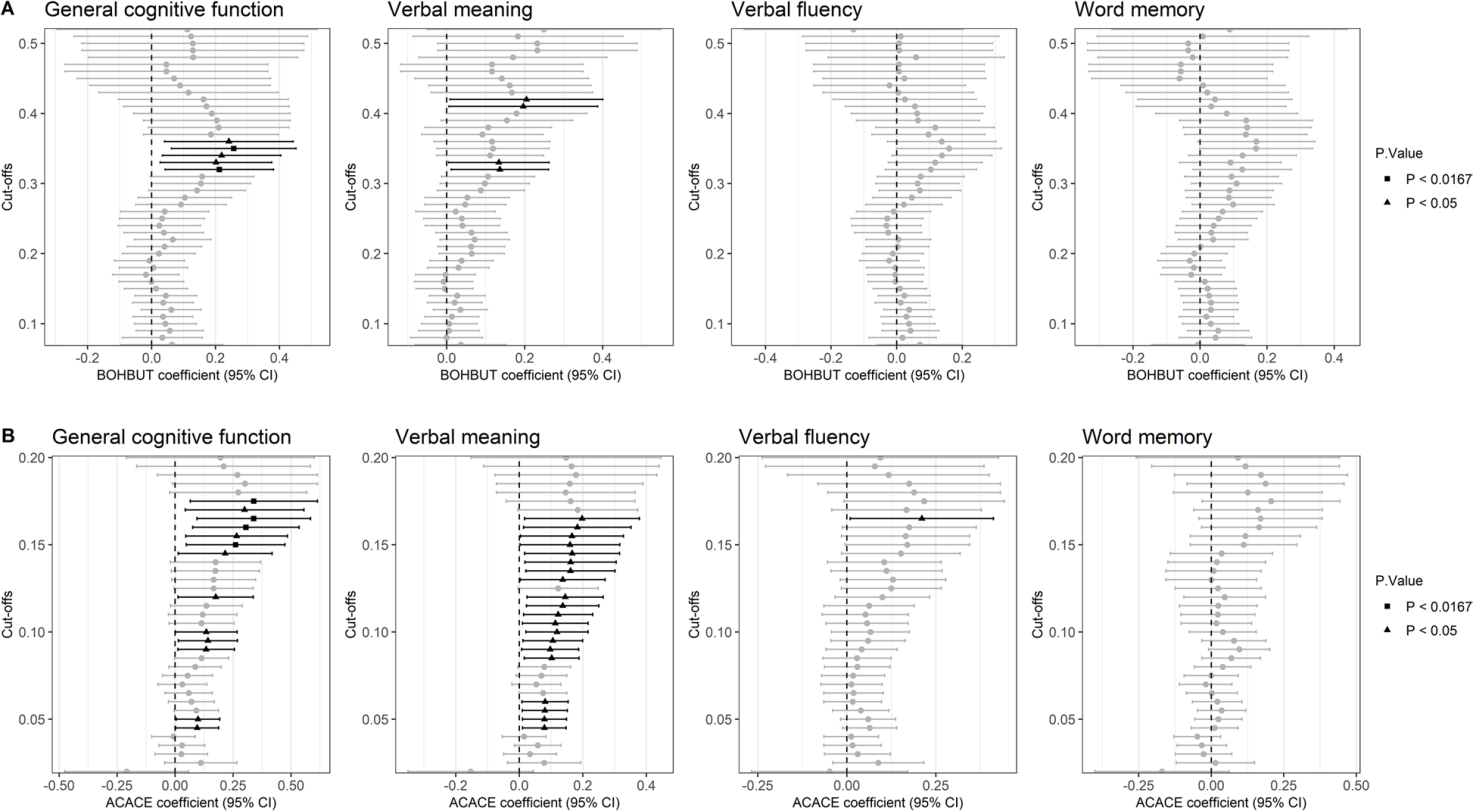
Observational analysis of ketone bodies 3-β-hydroxybutyrate (BOHBUT) and acetoacetate (ACACE) in relation to cognitive function. **A** Associations between observational BOHBUT and cognitive functions. **B** Associations between observational ACACE and cognitive functions. Ketone bodies (KBs) were categorized into two groups, e.g., high vs normal levels, based on the assumption that KBs could have a therapeutic benefit when the concentrations reach a certain level. The categorical cutoffs across the range of KBs were varied due to their unknown value. The full model was adjusted by age, sex, diabetes (yes/no), smoking (ever/never), alcohol consumption (heavy/other), waist-to-hip ratio, and occupational position (low/intermediate/high) across the cutoff values of BOHBUT from 0.07 to 0.52 mmol/L and ACACE from 0.02 to 0.20 mmol/L

Using regression analysis adjusted for age, sex, diabetes, smoking, alcohol consumption, waist-to-hip ratio, and occupational position with varying KB cutoffs, when BOHBUT reached 0.32 mmol/L, we observed strong evidence of association (*P* < 0.0167) between BOHBUT and general cognitive function (*β*_max_ = 0.257, *P* = 9.74 × 10^−3^ at the cutoff 0.35 mmol/L). For each cognitive function, we observed evidence of the suggestive association between BOHBUT and verbal meaning when BOHBUT reached 0.32 mmol/L (*β*_max_ = 0.136, *P* = 3.27 × 10^−2^ at the cutoff 0.32 mmol/L), while no evidence can be found in verbal fluency and word memory directly (Fig. 2).

For ACACE, a strong association between ACACE and general cognitive function was observed when ACACE reached 0.15 mmol/L (*β*_max_ = 3.40, *P* = 6.69 × 10^−3^ at the cutoff 0.165 mmol/L). For each cognitive function, we also found a suggestive association between ACACE and verbal meaning (*β*_max_ = 0.119, *P* = 1.77 × 10^−2^ at the cutoff 0.1 mmol/L). No clear evidence of association was observed in relation to verbal fluency and word memory (Fig. 2).

In addition, we performed association analyses between BOHBUT as a continuous variable and cognitive function scores with the same covariant adjustment. Little evidence of association was shown in general cognitive function (*β* = 0.318, *P* = 0.15), verbal meaning (*β* = 0.292, *P* = 7.28 × 10^−2^), verbal fluency (*β* = 0.097, *P* = 0.591), and word memory (*β* = 0.156, *P* = 0.412) (Additional file 1: Table S3).

Using ACACE as a continuous variable, positive effects were found in association analyses between ACACE and verbal meaning (*β* = 1.152, *P* = 4.59 × 10^−3^) and suggestive evidence between ACACE and general cognitive function (*β* = 1.285, *P* = 1.97 × 10^−2^). Little evidence can be observed in verbal fluency (*β* = 0.716, *P* = 0.113) and word memory (*β* = 0.253, *P* = 0.596) (Additional file 1: Table S3).

### Mendelian randomization study

#### Selection of instrumental variants

Table 1 presents the five independent selected SNPs associated with BOHBUT at a genome-wide significance level (*P* < 5 × 10^−8^). Using five instrumental SNPs for BOHBUT, the variance explained by genetics was 0.6%, and the *F*-statistic was 54.59. One of these SNPs is rs1508816, close to the well-known 3-oxoacid CoA transferase 1 (*OXCT1*) gene producing the key enzyme for ketone body catabolism. For ACACE, three independent SNPs were shown at a genome-wide significance level (Additional file 1: Table S4) with the variance explained by the genetics of 0.5% and the *F*-statistic of 72.24. For the suggestive set of instrumental variables (*P* < 1 × 10^−5^), 19 and 14 independent SNPs were identified for BOHBUT and ACACE, respectively (Additional file 1: Table S5 and S6).

**Table 1:** SNPs associated with 3-β-hydroxybutyrate under GWAS significance thresholds at *P* < 5 × 10^−8^

**SNPs**	**Chr**	**Closest reference gene**^a^	**Beta coefficient**	**SE**	***P*-value (discovery stage)**	**Effect allele**	**Effect allele frequency**
Association with *P* < 5 × 10^−8^							
rs9302635	16	*DHX38*	− 0.083	0.009	6.59 × 10^−21^	T	0.820
rs9987289	8	*PPP1R3B*	− 0.084	0.011	3.42 × 10^−14^	A	0.105
rs1508816	5	(*OXCT1*)	0.052	0.008	5.04 × 10^−11^	T	0.745
rs2419604	10	*GPAM*	− 0.050	0.008	4.27 × 10^−10^	A	0.287
rs6982502	8	(*TRIB1*)	0.041	0.007	2.25 × 10^−8^	T	0.540

Sample size = 45,031

*Chr* Chromosome, *SE* Standard error

^a^Genes for SNPs that are outside the transcript boundary of the protein-coding gene are shown in parentheses [e.g., (*OXCT1*)]

To assess instrument validity, we also performed association analyses between KB instrumental SNPs and potential confounders, including alcohol (heavy/other), diabetes (yes/no), waist-to-hip ratio, and occupational position (low/intermediate/high). After adjusting for Bonferroni correction (8 independent SNPs and 5 potential confounding factors), a significance threshold of 0.05/13 = 3.8 × 10^−3^ was applied. All instrumental SNPs were not associated with potential confounders, except for rs6982502, which was shown to be associated with smoking (ever/never) (*P*-value = 8.95 × 10^−5^) (Additional file 1: Table S7 and S8). For the robustness of results when MR assumptions are deviated, sensitivity analyses including weighted median and weighted mode methods were applied to ensure the reliability of our findings.

#### Causal effects of KBs on cognitive performance

**Fig. 3:**
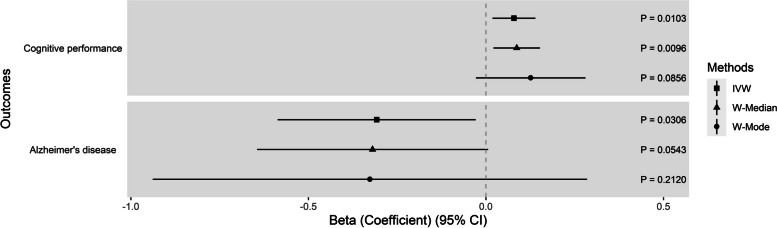
Causal estimates of 3-β-hydroxybutyrate on cognitive performance and Alzheimer’s disease from Mendelian randomization analyses. Causal estimates were calculated based on three MR methods: inverse-variance weighted (IVW), weighted median (W-Median), and weighted mode (W-Mode)

Using the strong set of instrumental variables, we observed a positive association of genetically predicted BOHBUT with cognitive performance using the IVW and W-median methods (IVW: *β*_IVW_ = 7.89 × 10^−2^, *P*_IVW_ = 1.03 × 10^−2^, W-median: *β*_W-Median_ = 8.65 × 10^−2^, *P*_W-Median_ = 9.60 × 10^−3^, W-mode: *β*_W-Mode_ = 0.126, *P*_W-Mode_ = 8.56 × 10^−2^) (Fig. 3, Additional file 1: Table S9). Evidence of causality between BOHBUT and cognitive performance was consistent by using a suggestive set of instrumental SNPs (*β*_IVW_ = 6.11 × 10^−2^, *P*_IVW_ = 2.03 × 10^−3^; *β*_W-Median_ = 5.68 × 10^−2^, *P*_W-Median_ = 2.30 × 10^−2^; *β*_W-Mode_ = 4.35 × 10^−2^, *P*_W-Mode_ = 0.367) (Additional file 1: Table S10). The Cochran’s *Q* test showed no evidence of heterogeneity (*P* > 0.1). In addition, no evidence of directional pleiotropy or outliers was observed using MR-Egger intercept (*P* > 0.05) and MR-PRESSO (*P* > 1 × 10^−6^) (Additional file 1: Table S11).

We did not find evidence of causality between ACACE and cognitive performance (Additional file 1: Table S12 and S13). Directional pleiotropic effects were not observed in the MR-Egger regression, and no outlier SNP was detected in the MR-PRESSO test (Additional file 1: Table S14).

#### Causal effects of KBs on Alzheimer’s disease

Using a strong set of instrumental variables, we observed some evidence for a protective effect of BOHBUT on AD using IVW (*β*_IVW_ =  − 0.308, odds ratio: OR = 0.735, *P*_IVW_ = 3.06 × 10^−2^). Similar causal estimates with less precision were shown using W-median (*β*_W-Median_ =  − 0.319, OR = 0.727, *P*_W-Median_ = 5.43 × 10^−2^) and W-mode (*β*_W-Mode_ =  − 0.327, OR = 0.721, *P*_W-Mode_ = 0.212) (Fig. 3). However, we did not observe evidence of causality under a suggestive set of instrumental SNPs (Additional file 1: Table S10). No significant causal association was observed between ACACE and AD (Additional file 1: Table S12 and S13), and evidence of directional pleiotropy and outliers was not observed (Additional file 1: Table S14).

After excluding rs6982503 previously shown an association with smoking, a potential confounding factor, the variance explained by genetics for BOHBUT was slightly reduced from 0.6 to 0.5%. The protective effect of BOHBUT on cognitive performance also remained (*β*_IVW_ = 9.26 × 10^−2^, *P*_IVW_ = 3.43 × 10^−3^; *β*_W-Median_ = 0.115, *P*_W-Median_ = 9.99 × 10^−4^), and a similar causal estimate of BOHBUT on AD was also observed, although with less precision (*β*_IVW_ =  − 0.274, OR = 0.76, *P*_IVW_ = 7.22 × 10^−2^). Evidence of neither directional pleiotropy nor outliers was observed using MR-Egger intercept and MR-PRESSO (Additional file 1: Table S15).

#### Causal effect estimation with cis-MR

The *OXCT1* gene encodes the enzyme Succinyl-CoA:3-ketoacid CoA transferase (SCOT), which is the key reaction that enables ketone body utilization for energy production [36]. We further performed *cis*-MR to investigate the therapeutic modulation of *OXCT1* as a partial predictor of KB. Three *cis*-acting SNPs (*P* < 1 × 10^−5^ and *r*^2^ < 0.4) were identified close to the *OXCT1* region with the variance explained of 0.19% and *F*-statistic of 28.40 (Additional file 1: Table S16). Accounting for the remaining residual correlation, *cis*-MR suggested little evidence of therapeutic modulation of *OXCT1* on either cognitive performance (*β*_IVW_ = 0.038, *P*_IVW_ = 0.312) or AD (*β*_IVW_ =  − 0.331, *P*_IVW_ = 0.290) while the direction of effect estimates was consistent with those previously shown (Additional file 1: Table S17). No evidence of pleiotropy was observed (*P* = 0.869).

## Discussion

Triangulating evidence from observational and MR studies supports a beneficial causal effect of BOHBUT on cognitive performance. First, an observational study showed that a range of increased levels of BOHBUT was associated with higher general cognitive function scores. Second, using a two-sample MR, BOHBUT showed evidence for causality on cognitive performance with a positive causal effect which was in concordance with the observational study previously observed. Using the MR study, we also observed the nominal protective effect of BOHBUT against AD, which supports the protective role of BOHBUT on cognitive function.

In clinical trials, either ketogenic diet or various types of KB supplement intakes were used to investigate the therapeutic effect on neurodegenerative diseases [24, 37]. The suggested therapeutic levels of KB for Alzheimer’s and epilepsy were 0.5 mmol/L and 2 mmol/L, respectively [24]; however, no investigation was shown for an early stage of cognitive decline. In our study, by varying KB cutoffs, we observed that BOHBUT was associated with the general cognitive performance score, when its concentration reached 0.32 mmol/L. Therefore, the lower concentration of KB could give a beneficial effect at an early stage.

In our observational analysis in which KB was categorized into two groups using exploratory therapeutic thresholds, we observed an attenuated estimate when the cutoff was increased. It was previously shown that a very high concentration of KBs (> 3 mmol/L) known as ketoacidosis [24] leads to delayed brain development [38] and increased risk of dementia in type 2 diabetes patients [39]. This may imply a non-linear relationship between KB and cognitive abilities. If this is the case, other possible assumptions on a therapeutic range of KB should be further explored. In our study, the KB range measured in WHII is limited to 0–1.5 mmol/L, with 98% of samples falling within a normal BOHBUT range below 0.5 mmol/L. Consequently, this limitation constrained our exploratory analysis within the framework of an observational study. Further investigation on a non-linear MR should be explored when individual patient data is available.

Clinical examinations using mental status tests, including word memory, verbal fluency, and verbal meaning, are recommended by current diagnostic guidelines to evaluate cognitive impairment. In this present study, we investigated the association of serum BOHBUT with the general cognitive function scores obtained from these mental status tests. These mental performances involve cognitive functions mainly in the prefrontal and left frontal cortices and hippocampus [40–42]. Perhaps further investigations are required to assess the causal role of KB on other parts of the brain, e.g., the occipital and parietal white matter that is myelinated region mediating network messaging and the processing speed [43, 44]. In addition, although we were less focused on ACACE due to its being an unstable compound [16], we still observed evidence of the association between increased ACACE and an improvement in general cognitive performance. This provided supportive evidence of what we previously observed in BOHBUT.

We further looked into downstream diseases from the progression of cognitive function decline. Unfortunately, a large GWAS for dementia was not publicly available; therefore, only AD was investigated. The protective causal effect of BOHBUT against AD was observed using IVW, and similar causal effects with less precision were shown using W-median and W-mode. This nominal protective effect gives some supportive evidence for a beneficial effect of BOHBUT on cognitive performance previously found. MR uses genetic variants as a natural experiment which is less prone to bias from confounding factors and reverse causation compared to an observational analysis, and thus, MR infers stronger evidence for causality.

Using genetic variants as an instrument, our work suggests that lifelong naturally elevated KBs have a beneficial effect on cognitive performance and, considering the protective causal role of BOHBUT, on AD. This concept might align with the beneficial effect of consuming a low-carbohydrate diet and perhaps coincide with the concept of adhering to set mealtime schedules, such as intermittent fasting, which was previously suggested to yield cognitive benefits [45]. It is important to emphasize that findings from our observational and genetic analyses do not inform about the potential immediate benefits of interventions aimed at elevating KB levels on cognitive performance. Further RCTs are needed to investigate these short-term effects. If similar outcomes are replicated in RCTs, short-term therapeutic interventions, like the adoption of a ketogenic diet or the use of ketone supplements, could be considered as a combined therapeutic strategy.

Our study provides a number of strengths that reinforce the validity of the findings. Firstly, KBs were measured using NMR spectroscopy, which is an advanced analytical technique allowing for the identification and precise quantification of metabolites. Secondly, the concordance of the effect direction in triangulating evidence from observational and MR studies strengthens the causal evidence observed between KBs and cognitive functions. In addition, one of the SNPs discovered in our in-house meta-analysis for KB is located near *OXCT1*, which is known to be in the pathway for KB catabolism [46]. Therefore, this partially reassures us that selected instrumental SNPs reflect an underlying mechanism of KB and as a consequence, a good proxy as an instrument variable.

Our study also has limitations that should be taken into consideration. Firstly, due to the challenge to control the fasting period before blood collection, there is a potential for measurement error. Higher levels of KB can be measured if individuals have longer fasting periods. This may affect the identification of the underlying KB SNPs that were used as instruments in MR analyses, as well as the results observed in our observational study. Secondly, our instrumental SNPs provide limited variance explained, i.e., 0.6% for BOHBUT and 0.5% for ACACE. Further discovery of underlying KB SNPs is required in the study with larger sample sizes. Thirdly, as mentioned above, we assumed a linear relationship between KB and cognitive performance. If this is not the case, the linear model would have a limited ability to capture nonlinear complexities in the data and may result in biased estimates. Lastly, our study is based on a sample consisting of Caucasian individuals; therefore, caution should be taken when generalizing the finding to other populations.

## Conclusions

Using triangulating evidence from observational and MR studies, we observed an association and some evidence of a causal effect of BOHBUT on cognitive performance. Our study suggested that increased BOHBUT has a beneficial effect on cognitive performance. In addition, when considering the causal role of BOHBUT on AD, some protective causal effect was observed against this neurodegenerative disease. Our findings are consistent with a hypothesis that increased BOHBUT may improve cognitive function, contributing to decreased progression of cognitive impairment and risk of AD.

### Supplementary Information

**Additional file 1:** **Appendix S1.** List of additional UCLEB members. **Supplementary Methods.** **Fig. S1**. Hypothesized biological pathway linking ketone body metabolism and cognitive functions. During fasting, fatty acids are converted into acetoacetate (ACACE) and 3-β-hydroxybutyrate (BOHBUT) through Acetyl CoA. BOHBUT and ACACE cross the blood-brain barrier (BBB) and enter neurons through a monocarboxylate transporter (MCT) channel. Once BOHBUT and ACACE enter the brain, a series of reactions occur to form Acetyl CoA. These reactions involve the 3-oxoacid CoA-transferase 1 (OXCT1) enzyme which is encoded by the OXCT1 gene. The product of OXCT1 is then converted to acetyl-CoA and subsequently enters the tricarboxylic acid (TCA) cycle for oxidation and ATP production [5]. BOHBUT and ACACE have been suggested to contribute to the secretion of Brain-derived neurotrophic factor (BDNF). This protein molecule is involved in the enhancement of mitochondrial biogenesis and synaptic plasticity, a key to learning ability and memory [6,7]. **Table S1. **Details of studies and datasets included in analyses. **Table S2.**Baseline characteristics of participants in the WHII study. **Table S3. **Multiple linear regression examining the association between BOHBUT and ACACE as a continuous variable and cognitive function scores. The models were adjusted by age, sex, diabetes (yes/no), smoking (ever/never), alcohol consumption (heavy/other), waist-to-hip ratio, and socioeconomic status (low/intermediate/high). **Table S4. **SNPs associated with ACACE using clumping windows -/+ 10 000 kb and *r*^2^ < 0.001. **Table S5. **SNPs associated with BOHBUT using clumping window -/+ 10 000 kb and r^2^< 0.001. **Table S6. **SNPs associated with ACACE using clumping window -/+ 10 000 kb and *r*^2^< 0.001. **Table S7. **Association analyses between 3-β-hydroxybutyrate instrumental SNPs and potential confounders, including alcohol (ever/never), diabetes (yes/no), adiposity, i.e., waist-to-hip ratio, and occupational position (low/intermediate/high). **Table S8. **Association analyses between acetoacetate instrumental SNPs and potential confounders, including alcohol (ever/never), diabetes (yes/no), adiposity, i.e., waist-to-hip ratio, and occupational position (low/intermediate/high). **Table S9. **MR of BOHBUT on cognitive performance and Alzheimer’s disease using 5 instrumental SNPs associated with BOHBUT (GWAS threshold: *P*< 5 × 10^-8^). **Table S10. **MR of BOHBUT on cognitive performance and Alzheimer’s disease using 19 instrumental SNPs associated with BOHBUT (GWAS threshold: *P* < 1 × 10^-5^).**Table S11. Heterogeneity** and pleiotropy tests in MR of BOHBUT on cognitive performance and Alzheimer’s disease. **Table S12.** MR of ACACE on cognitive performance and Alzheimer’s disease using 3 instrumental SNPs associated with ACACE (GWAS threshold: *P* < 5 × 10^-8^). **Table S13.** MR of ACACE on cognitive performance and Alzheimer’s disease using 14 instrumental SNPs associated with ACACE (GWAS threshold: *P* < 1 × 10^-5^). **Table S14.** Heterogeneity and pleiotropy tests in MR of ACACE on cognitive performance and Alzheimer’s disease. **Table S15.** MR of BOHBUT on cognitive performance and Alzheimer’s disease using 4 instrumental SNPs (rs6982503 was removed) associated with BOHBUT (GWAS threshold: *P* < 5 × 10^-8^). **Table S16.** *cis*-SNPs in OXCT1 (GWAS threshold: *P* < 1 × 10^-5^) using clumping window -/+ 10 000 kb and LD-*r*^2^ < 0.4. **Table S17.** *cis*-MR for a causal estimate of OXCT1 locus on cognitive performance and Alzheimer’s disease.

## Data Availability

Data from the UCLEB Consortium is available upon request. GWAS summary statistics used and/or analyzed in this study are publicly available and accessible (Additional file 1: Table S1).

## Abbreviations

ACACE: Acetoacetate
AD: Alzheimer’s disease
Aβ: β-Amyloid
BDNF: Brain-derived neurotrophic factor
BOHBUT: 3-β-Hydroxybutyrate
GWAS: Genome-wide association studies
IGAP: International Genomics of Alzheimer’s Project
IQR: Interquartile range
IVW: Inverse variance weighted
KBs: Ketone bodies
MR: Mendelian randomization
MR-PRESSO: Mendelian Randomization Pleiotropy RESidual Sum and Outlier
NMR: Nuclear magnetic resonance
OR: Odds ratio
OXCT1: 3-Oxoacid CoA transferase 1
RCTs: Randomized controlled trials
SCOT: Succinyl-CoA:3-ketoacid CoA transferase
SNPs: Single nucleotide polymorphisms
SSGAC: Social Science Genetic Association Consortium
UCLEB: University College London-London School of Hygiene and Tropical Medicine-Edinburgh-Bristol
WHII: Whitehall II study
W-Median: Weighted median method
W-Mode: Weighted mode-based estimation method
