# Supplementary material for: Triangulating evidence from observational and Mendelian randomization studies of ketone bodies for cognitive performance
Source: BMC Med. 2023 Sep 4;21:340. doi: 10.1186/s12916-023-03047-7 (PMC10478491; doi:10.1186/s12916-023-03047-7)
Supplement: Supplementary file 1 — Additional file 1: Appendix S1. List of additional UCLEB members. Supplementary Methods. Fig. S1. Hypothesized biological pathway linking ketone body metabolism and cognitive functions. During fasting, fatty acids are converted into acetoacetate (ACACE) and 3-β-hydroxybutyrate (BOHBUT) through Acetyl CoA. BOHBUT and ACACE cross the blood-brain barrier (BBB) and enter neurons through a monocarboxylate transporter (MCT) channel. Once BOHBUT and ACACE enter the brain, a series of reactions occur to form Acetyl CoA. These reactions involve the 3-oxoacid CoA-transferase 1 (OXCT1) enzyme which is encoded by the OXCT1 gene. The product of OXCT1 is then converted to acetyl-CoA and subsequently enters the tricarboxylic acid (TCA) cycle for oxidation and ATP production [5]. BOHBUT and ACACE have been suggested to contribute to the secretion of Brain-derived neurotrophic factor (BDNF). This protein molecule is involved in the enhancement of mitochondrial biogenesis and synaptic plasticity, a key to learning ability and memory [6,7]. Table S1. Details of studies and datasets included in analyses. Table S2.Baseline characteristics of participants in the WHII study. Table S3. Multiple linear regression examining the association between BOHBUT and ACACE as a continuous variable and cognitive function scores. The models were adjusted by age, sex, diabetes (yes/no), smoking (ever/never), alcohol consumption (heavy/other), waist-to-hip ratio, and socioeconomic status (low/intermediate/high). Table S4. SNPs associated with ACACE using clumping windows -/+ 10 000 kb and r2 < 0.001. Table S5. SNPs associated with BOHBUT using clumping window -/+ 10 000 kb and r2< 0.001. Table S6. SNPs associated with ACACE using clumping window -/+ 10 000 kb and r2< 0.001. Table S7. Association analyses between 3-β-hydroxybutyrate instrumental SNPs and potential confounders, including alcohol (ever/never), diabetes (yes/no), adiposity, i.e., waist-to-hip ratio, and occupational position (low/intermediat [file 12916_2023_3047_MOESM1_ESM.docx]

**Additional file 1:**

**Triangulating evidence from observational and Mendelian randomization studies of ketone bodies for cognitive performance**

**Table of Contents**

Appendix S1 List of additional UCLEB members 2

Supplementary Method 4

Supplementary Figures 5

Supplementary Tables 6

References 13

## ****Appendix S1**** List of additional UCLEB members

| **Author** | **Affiliation** |
| --- | --- |
| Aleksandra Gentry‐Maharaj | MRC Clinical Trials Unit at UCL, Institute of Clinical Trials & Methodology, University College London, London, United Kingdom |
| Alun D. Hughes | MRC Unit for Lifelong Health and Ageing at UCL, Department of Population Science & Experimental Medicine, Institute of Cardiovascular Science, Faculty of Population Health Sciences, University College London, London, United Kingdom |
| Ann Walker | Centre for Cardiovascular Genetics, Dept. of Medicine, University College London, London, United Kingdom |
| Barbara Jefferis | UCL Department of Primary Care & Population Health, UCL Medical School, London United Kingdom |
| Caroline Dale | Farr Institute of Health Informatics, University College London, London, United Kingdom |
| Chris Finan | Institute of Cardiovascular Science, University College London, London, United Kingdom |
| Christine Power | MRC Centre of Epidemiology for Child Health, Department of Population Health Sciences, UCL Institute of Child Health, London, United Kingdom |
| Claudia Langenberg | Department of Epidemiology & Public Health, UCL Institute of Epidemiology & Health Care, University College London, London, United Kingdom |
| Deborah A Lawlor | 1) MRC Integrative Epidemiology Unit at the University of Bristol, Bristol, United Kingdom  2) Population Health, Bristol Medical School, University of Bristol, Bristol, United Kingdom 3) Bristol NIHR Bristol Biomedical Research Centre, University Hospitals Bristol National Health Service Foundation Trust and University of Bristol, Bristol, United Kingdom |
| Diana Kuh | MRC Unit for Lifelong Health and Ageing, London, United Kingdom |
| Elina Hypponen | MRC Centre of Epidemiology for Child Health, Department of Population Health Sciences, UCL Institute of Child Health, University College London, London, United Kingdom |
| Fotios Denros | 1) Institute of Cardiovascular Science, Faculty of Population Health, University College London, London, United Kingdom 2) Department of Life Sciences, College of Health and Life Sciences, Brunel University London, Uxbridge, United Kingdom |
| Frank Dudbridge | Department of Health Sciences, Centre for Medicine, University of Leicester, Leicester, United Kingdom |
| Ghazaleh Fatemifar | Farr Institute of Health Informatics, University College London, London, United Kingdom |
| Goya Wannamethee | Primary Care and Population Health, University College London, London, United Kingdom |
| John Whittaker | Genetics Division, Research and Development, GlaxoSmithKline, Harlow, United Kingdom |
| Juan Pablo Casas | Massachusetts Veterans Epidemiology Research and Information Center (MAVERIC), VA Boston Healthcare System, Boston MA, USA |
| Maria Carolina Borges | 1) MRC Integrative Epidemiology Unit at the University of Bristol, Bristol, United Kingdom 2) Population Health Sciences, Bristol Medical School, University of Bristol, Bristol, United Kingdom |
| María Gordillo-Marañón | Institute of Cardiovascular Science, Faculty of Population Health, University College London, London, United Kingdom |
| Meena Kumari | 1) Department of Epidemiology and Public Health, University College London, London, United Kingdom 2) Institute for Social and Economic Research, University of Essex, United Kingdom |
| Nishi Chaturvedi | Institute of Cardiovascular Science, University College London, London, United Kingdom |
| Peter Whincup | Population Health Research Institute, St George’s, University of London, United Kingdom |
| Philippa Talmud | Centre for Cardiovascular Genetics, Dept. of Medicine, University College London, London, United Kingdom |
| Reecha Sofat | Centre for Clinical Pharmacology, University College London, London, United Kingdom |
| Rui Providencia | 1) Farr Institute of Health Informatics, University College London, London, United Kingdom 2) Barts Heart Centre, St Bartholomew's Hospital, Barts Health NHS Trust, London, United Kingdom |
| S. Goya Wannamethee | UCL Department of Primary Care & Population Health, UCL Medical School, London, United Kingdom |
| Stela McLachlan | Centre for Population Health Sciences, The Usher Institute of Population Health Sciences and Informatics, University of Edinburgh, United Kingdom |
| Steve Humphries | Centre for Cardiovascular Genetics, Dept. of Medicine, University College London, London, United Kingdom |
| Therese Tillin | Cardiometabolic Phenotyping Group, Institute of Cardiovascular Science, University College London, London, United Kingdom |
| Usha Menon | MRC Clinical Trials Unit at UCL, Institute of Clinical Trials & Methodology, University College London, London, United Kingdom |
| Victoria Garfield | Department of Epidemiology & Public Health, UCL Institute of Epidemiology & Health Care, University College London, London, United Kingdom |
| Vincent Plagnol | University College London Genetics Institute, Department of Genetics, Environment and Evolution, London, United Kingdom |
| Yoav Ben‐Shlomo | Population Health Sciences, Bristol Medical School, University of Bristol, Bristol, United Kingdom |

## ****Supplementary Method****

*The Whitehall II (WHII) study: Details of dataset*

*Blood sample collection*

5,506 individuals (72% men) were recruited at ages 44 to 70 years. Fasting venous blood samples were collected, centrifuged, and serum was stored in aliquots at -80 °C prior to metabolic profiling by serum nuclear magnetic resonance (NMR) metabolomics (1).

*The NMR metabolomics platform and quality control*

WHII used Nightingale Health's metabolic biomarker platform (2). A total of 233 metabolic biomarkers were quantified as part of the Consortium of Metabolomic studies. The biomarkers include detailed measures of cholesterol metabolism, fatty acid compositions, and various low-molecular-weight metabolites, such as amino acids, ketone bodies, and glycolysis metabolites. The majority of the biomarkers are measured in absolute concentration units (mmol/L).

For quality control, the platform processes data automatically and executes quality control procedures reporting degradation and contamination issues. If the concentration of metabolite is above the limit of detection but below the limit of quantification due to biological reasons or interference from external compounds, the metabolite value is set to 0. Metabolites reported in WHII underwent this strict quality control and any outlier values in metabolite concentrations (≥±9 SD) have been excluded (1).

*In-house KBs meta-analysis between the UCLEB consortium (3) and Kettunen et al. (4)*

In the GWAS by Kettunen et al, the analysis was performed on 14 cohort studies of up to 24,925 individuals for 123 direct and derived circulating metabolic measures. All metabolites were first adjusted for age, sex, and 10 first principal components from genomic data and the resulting residuals were transformed to a normal distribution by inverse rank-based normal transformation. Similarly, in UCLEB, the GWAS analysis was performed on 8 studies of up to 20,106 individuals for 230 direct and derived circulating metabolic measures. All metabolites were transformed using the inverse rank-based normal transformation and tested against genotypes adjusting for age and sex. The fixed-effect meta-analysis of publicly available summary statistics in Kettunen et al and summary statistics in the UCLEB consortium was then performed to obtain a sample size of up to 45,031 individuals. Participants were almost exclusively of European ancestry.

## ****Supplementary Figures****


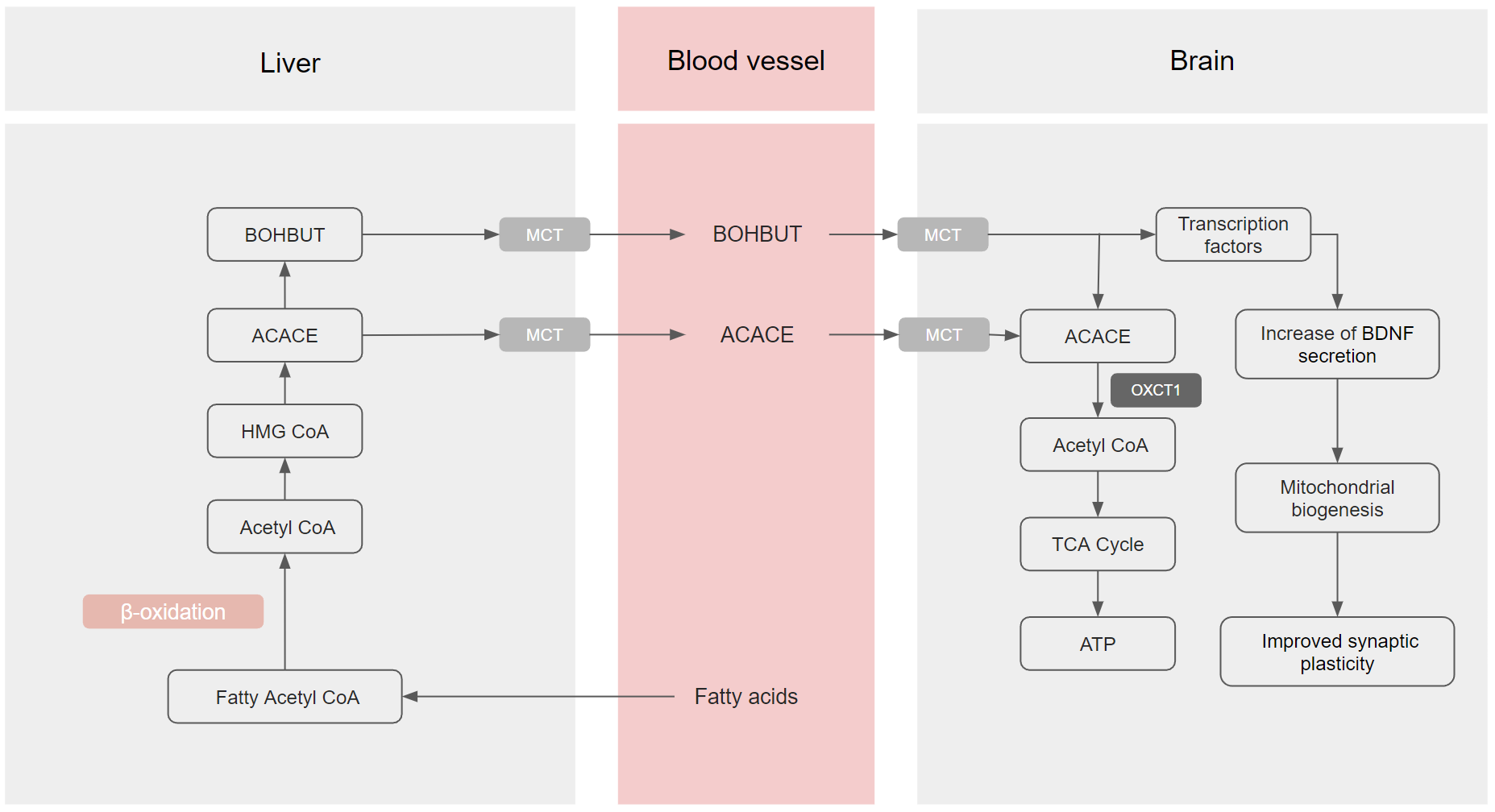
**Fig. S1 Hypothesized biological pathway linking ketone body metabolism and cognitive functions.** During fasting, fatty acids are converted into acetoacetate (ACACE) and 3-β-hydroxybutyrate (BOHBUT) through Acetyl CoA. BOHBUT and ACACE cross the blood-brain barrier (BBB) and enter neurons through a monocarboxylate transporter (MCT) channel. Once BOHBUT and ACACE enter the brain, a series of reactions occur to form Acetyl CoA. These reactions involve the 3-oxoacid CoA-transferase 1 (*OXCT1*) enzyme which is encoded by the *OXCT1* gene. The product of *OXCT1* is then converted to acetyl-CoA and subsequently enters the tricarboxylic acid (TCA) cycle for oxidation and ATP production (5). BOHBUT and ACACE have been suggested to contribute to the secretion of Brain-derived neurotrophic factor (BDNF). This protein molecule is involved in the enhancement of mitochondrial biogenesis and synaptic plasticity, a key to learning ability and memory (6, 7).

## ****Supplementary Tables****

**Table S1** Details of studies and datasets included in analyses.

| **Risk factor / outcome** | **Participants (N cases)** | **Consortium / study** | **Ethnicity** | **Covariates adjusted in GWAS** | **PMID (Web source if publicly available)** |
| --- | --- | --- | --- | --- | --- |
| **Observational study** | | | | | |
| BOHBUT | 4,621 | UCLEB (WHII) | European | Age and sex | 23977022 |
| ACACE | 4,637 |  |  |  |  |
| Word memory | 3,115 |  |  |  |  |
| Verbal fluency | 3,122 |  |  |  |  |
| Verbal meaning | 3,142 |  |  |  |  |
| **Mendelian randomization study (Exposures)** | | | | | |
| BOHBUT | 33,029 | UCLEB and  Kettunen et al. | European | Age and sex | 23977022 and 27005778 |
| ACACE | 33,029 |  |  |  |  |
| **Mendelian randomization study (Outcomes)** | | | | | |
| Cognitive performance | 257,841 | SSGAC | European | Year of birth, sex, sex*year of birth, and the first 10 PCs | 30038396 |
| Alzheimer’s disease | 54,162 (17,008) | IGAP | Mixed | Age, sex and PCs | 24162737 (http://web.pasteur-lille.fr/en/recherche/u744/igap/igap_download.php) |

GWAS, Genome-wide association study; PubMed ID, PubMed identifier; BOHBUT, 3-β-hydroxybutyrate; ACACE, Acetoacetate; PC, principal components; UCLEB, University College-London School-Edinburgh-Bristol consortium; WHII, Whitehall II study; SSGAC, Social Science Genetic Association Consortium; IGAP, International Genomics of Alzheimer’s Project.

**Table S2** Baseline characteristics of participants in the WHII study.

| **Characteristics** | **WHII** |
| --- | --- |
| Participants, n | 5,506 |
| Age (mean, SD) | 55.56 (5.947) |
| Sex (men, %) | 72.14 |
| Diabetes (yes, %) | 9.37 |
| Smoking (yes, %) | 16.56 |
| Alcohol consumption (heavy, other) |  |
| 1: Heavy (> 14 units/week, yes, %) | 35.34 |
| 2: Other (≤ 14 units/week, yes, %) | 64.66 |
| Waist-to-hip ratio (mean, SD) | 0.89 (0.0860) |
| Occupational position |  |
| 1: Senior administrative grades (yes, %) | 50.38 |
| 2: Middle-ranking executive grades (yes, %) | 15.22 |
| 3: Clerical and office support grades (yes, %) | 34.40 |
| **Ketone Bodies (median, IQR)** |  |
| BOHBUT (mmol/L) | 0.116 (0.092) |
| ACACE (mmol/L) | 0.044 (0.043) |

WHII, Whitehall II study; SD, standard deviation; BOHBUT, 3-β-hydroxybutyrate; ACACE, Acetoacetate;
IQR, Interquartile range.

**Table S3** Multiple linear regression examining the association between BOHBUT and ACACE as a continuous variable and cognitive function scores. The models were adjusted by age, sex, diabetes (yes/no), smoking (ever/never), alcohol consumption (heavy/other), waist-to-hip ratio, and socioeconomic status (low/intermediate/high).

| **Selected covariates** | **BOHBUT** | | **ACACE** | |
| --- | --- | --- | --- | --- |
|  | **Beta Coefficient (SE)** | **P-value** | **Beta Coefficient (SE)** | **P-value** |
| **General cognitive function** |  |  |  |  |
| Sex, Age | 0.61 (0.25) | 1.47E-02** | 2.219 (0.623) | 3.72E-04** |
| Sex, Age, DM | 0.599 (0.249) | 1.62E-02** | 2.202 (0.621) | 4.00E-04** |
| Sex, Age, DM, Smoke, Alcohol | 0.551 (0.247) | 2.62E-02* | 1.954 (0.618) | 1.59E-03** |
| Sex, Age, DM, Smoke, Alcohol, WHR | 0.49 (0.247) | 4.68E-02* | 1.776 (0.616) | 3.99E-03** |
| Sex, Age, DM, Smoke, Alcohol, WHR, Occupational position | 0.318 (0.22) | 1.50E-01 | 1.285 (0.551) | 1.97E-02* |
| **Verbal Meaning** |  |  |  |  |
| Sex, Age | 0.524 (0.187) | 5.15E-03** | 1.892 (0.467) | 5.19E-05** |
| Sex, Age, DM | 0.518 (0.187) | 5.61E-03** | 1.882 (0.466) | 5.57E-05** |
| Sex, Age, DM, Smoke, Alcohol | 0.481 (0.186) | 9.72E-03** | 1.694 (0.464) | 2.62E-04** |
| Sex, Age, DM, Smoke, Alcohol, WHR | 0.429 (0.185) | 2.03E-02* | 1.542 (0.462) | 8.49E-04** |
| Sex, Age, DM, Smoke, Alcohol, WHR, Occupational position | 0.292 (0.163) | 7.28E-02 | 1.152 (0.406) | 4.59E-03** |
| **Verbal fluency** |  |  |  |  |
| Sex, Age | 0.26 (0.193) | 1.79E-01 | 1.248 (0.482) | 9.73E-03** |
| Sex, Age, DM | 0.255 (0.193) | 1.88E-01 | 1.239 (0.482) | 1.02E-02** |
| Sex, Age, DM, Smoke, Alcohol | 0.229 (0.193) | 2.35E-01 | 1.091 (0.481) | 2.33E-02* |
| Sex, Age, DM, Smoke, Alcohol, WHR | 0.201 (0.193) | 2.97E-01 | 1.01 (0.481) | 3.59E-02* |
| Sex, Age, DM, Smoke, Alcohol, WHR, Occupational position | 0.097 (0.181) | 5.91E-01 | 0.716 (0.452) | 1.13E-01 |
| **Word Memory** |  |  |  |  |
| Sex, Age | 0.249 (0.193) | 1.97E-01 | 0.54 (0.482) | 2.62E-01 |
| Sex, Age, DM | 0.243 (0.193) | 2.08E-01 | 0.529 (0.481) | 2.71E-01 |
| Sex, Age, DM, Smoke, Alcohol | 0.224 (0.193) | 2.45E-01 | 0.449 (0.482) | 3.51E-01 |
| Sex, Age, DM, Smoke, Alcohol, WHR | 0.202 (0.193) | 2.95E-01 | 0.383 (0.482) | 4.27E-01 |
| Sex, Age, DM, Smoke, Alcohol, WHR, Occupational position | 0.156 (0.191) | 4.12E-01 | 0.253 (0.477) | 5.96E-01 |

BOHBUT, β-hydroxybutyrate; ACACE, Acetoacetate; SE, standard error; DM, diabetes

**Table S4** SNPs associated with ACACE using clumping windows -/+ 10 000 kb and r^2^ < 0.001.

| **SNPs** | **Chr** | **Closest  Reference Gene*** | **Beta Coefficient** | **SE** | **P-value  (Discovery Stage)** | **Effect Allele** | **Effect Allele Frequency** |
| --- | --- | --- | --- | --- | --- | --- | --- |
| Association with P < 5 x 10^-8^ | | | | | | | |
| rs964184 | 11 | *ZPR1* | -0.099 | 0.011 | 2.51E-21 | C | 0.861 |
| rs2169387 | 8 | *PPP1R3B* | -0.085 | 0.012 | 1.07E-13 | A | 0.117 |
| rs1508816 | 5 | (*OXCT1*) | 0.061 | 0.008 | 4.72E-13 | T | 0.737 |

SNPs, single-nucleotide polymorphisms; Chr, chromosome; SE, standard error; Sample size = 45,031; R^2^ = 0.005; F-statistic = 72.24.
*Genes for SNPs that are outside the transcript boundary of the protein-coding gene are shown in parentheses [e.g., (*OXCT1*)].

**Table S5** SNPs associated with BOHBUT using clumping window -/+ 10 000 kb and r^2^ < 0.001.

| **SNPs** | **Chr** | **Closest  Reference Gene*** | **Beta Coefficient** | **SE** | **P-value  (Discovery Stage)** | **Effect Allele** | **Effect Allele Frequency** |
| --- | --- | --- | --- | --- | --- | --- | --- |
| Association with P < 5 × 10^-8^ | | | | | | | |
| rs9302635 | 16 | *DHX38* | -0.083 | 0.009 | 6.59E-21 | T | 0.820 |
| rs9987289 | 8 | *PPP1R3B* | -0.084 | 0.011 | 3.42E-14 | A | 0.105 |
| rs1508816 | 5 | (*OXCT1*) | 0.052 | 0.008 | 5.04E-11 | T | 0.745 |
| rs2419604 | 10 | *GPAM* | -0.050 | 0.008 | 4.27E-10 | A | 0.287 |
| rs6982502 | 8 | (*TRIB1*) | 0.041 | 0.007 | 2.25E-08 | T | 0.540 |
| Association with 5 × 10^-8^ < P < 1 × 10^-5^ | | | | | | | |
| rs72999138 | 3 | (*PLSCR4*) | -0.073 | 0.014 | 2.38E-07 | T | 0.922 |
| rs863003 | 1 | *ACKR1* | -0.042 | 0.008 | 3.08E-07 | A | 0.771 |
| rs6886903 | 5 | *IQGAP2* | 0.044 | 0.009 | 8.64E-07 | A | 0.221 |
| rs4778599 | 15 | *ARNT2* | 0.036 | 0.008 | 2.12E-06 | A | 0.352 |
| rs10864726 | 1 | *GALNT2* | -0.033 | 0.007 | 2.92E-06 | T | 0.594 |
| rs205752 | 7 | *LINC-PINT* | -0.034 | 0.007 | 2.98E-06 | A | 0.431 |
| rs1601358 | 2 | (*LOC107985826*) | -0.034 | 0.007 | 3.54E-06 | A | 0.642 |
| rs9668178 | 12 | *SSPN* | 0.037 | 0.008 | 4.59E-06 | A | 0.248 |
| rs35716369 | 13 | (*ARHGEF7*) | -0.048 | 0.011 | 6.09E-06 | T | 0.131 |
| rs62579883 | 9 | (*LOC124902266*) | -0.111 | 0.025 | 6.27E-06 | A | 0.030 |
| rs2587416 | 18 | (*CDH7*) | -0.032 | 0.007 | 8.26E-06 | A | 0.500 |
| rs141346935 | 7 | *HIBADH* | 0.149 | 0.033 | 8.51E-06 | T | 0.017 |
| rs2740488 | 9 | *ABCA1* | 0.038 | 0.009 | 9.16E-06 | A | 0.775 |
| rs7161231 | 14 | (*SERPINA6*) | 0.055 | 0.012 | 9.97E-06 | T | 0.119 |

SNPs, single-nucleotide polymorphisms; Chr, chromosome; SE, standard error; Sample size = 45,031; R^2^ = 0.013; F-statistic = 31.61.

*Genes for SNPs that are outside the transcript boundary of the protein-coding gene are shown in parentheses [e.g., (*OXCT1*)].

**Table S6** SNPs associated with ACACE using clumping window -/+ 10 000 kb and r^2^ < 0.001.

| **SNPs** | **Chr** | **Closest  Reference Gene*** | **Beta Coefficient** | **SE** | **P-value  (Discovery Stage)** | **Effect Allele** | **Effect Allele Frequency** |
| --- | --- | --- | --- | --- | --- | --- | --- |
| Association with P < 5 × 10^-8^ | | | | | | | |
| rs964184 | 11 | *ZPR1* | -0.099 | 0.011 | 2.51E-21 | C | 0.861 |
| rs2169387 | 8 | *PPP1R3B* | -0.085 | 0.012 | 1.07E-13 | A | 0.117 |
| rs1508816 | 5 | (*OXCT1*) | 0.061 | 0.008 | 4.72E-13 | T | 0.737 |
| Association with 5 × 10^-8^ < P < 1 × 10^-5^ | | | | | | | |
| rs2281718 | 1 | *GALNT2* | 0.038 | 0.008 | 8.11E-07 | A | 0.408 |
| rs1871554 | 12 | *SSPN* | 0.053 | 0.011 | 1.82E-06 | T | 0.876 |
| rs2250509 | 1 | *MYBPH* | -0.058 | 0.012 | 1.84E-06 | A | 0.136 |
| rs78569940 | 1 | *AK5* | -0.096 | 0.020 | 2.26E-06 | A | 0.045 |
| rs7208828 | 17 | (*LOC105371750*) | -0.037 | 0.008 | 5.16E-06 | A | 0.354 |
| rs76334369 | 11 | (*OR5AK1P*) | -0.129 | 0.028 | 5.40E-06 | A | 0.977 |
| rs62023507 | 15 | *ONECUT1* | -0.082 | 0.018 | 6.16E-06 | A | 0.945 |
| rs11943376 | 4 | *MAPK10* | -0.051 | 0.012 | 7.40E-06 | T | 0.135 |
| rs149468507 | 8 | (*MSRA*) | 0.111 | 0.025 | 8.12E-06 | A | 0.040 |
| rs309126 | 2 | (*MCM6*) | -0.051 | 0.011 | 8.87E-06 | T | 0.872 |
| rs1494345 | 9 | *FREM1* | 0.034 | 0.008 | 9.85E-06 | T | 0.473 |

SNPs, single-nucleotide polymorphisms; Chr, chromosome; SE, standard error; Sample size = 45,031; R^2^ = 0.011; F-statistic = 35.80.

*Genes for SNPs that are outside the transcript boundary of the protein-coding gene are shown in parentheses [e.g., (*OXCT1*)].

**Table S7** Association analyses between 3-β-hydroxybutyrate instrumental SNPs and potential confounders, including alcohol (ever/never), diabetes (yes/no), adiposity, i.e., waist-to-hip ratio, and occupational position (low/intermediate/high).

| **SNPs** | **Beta Coefficient** | **SE** | **P-value** |
| --- | --- | --- | --- |
| **Diabetes (yes/no)** | | | |
| rs9302635 | 0.002 | 0.004 | 5.09E-01 |
| rs9987289 | 0.012 | 0.005 | 1.02E-02 |
| rs1508816 | 0.003 | 0.003 | 3.54E-01 |
| rs2419604 | 0.003 | 0.003 | 3.77E-01 |
| rs6982502 | -0.002 | 0.003 | 5.31E-01 |
| **Smoking (ever/never)** | | | |
| rs9302635 | 0.008 | 0.010 | 3.89E-01 |
| rs9987289 | 0.020 | 0.013 | 1.06E-01 |
| rs1508816 | -0.012 | 0.009 | 1.98E-01 |
| rs2419604 | -0.001 | 0.008 | 9.23E-01 |
| rs6982502 | -0.028 | 0.007 | 8.95E-05 |
| **Alcohol consumption (heavy/other)** | | | |
| rs9302635 | -0.011 | 0.016 | 4.94E-01 |
| rs9987289 | 0.001 | 0.021 | 9.49E-01 |
| rs1508816 | -0.016 | 0.015 | 3.01E-01 |
| rs2419604 | -0.003 | 0.014 | 8.51E-01 |
| rs6982502 | -0.008 | 0.012 | 5.03E-01 |
| **Waist-to-Hip Ratio** | | | |
| rs9302635 | -0.004 | 0.002 | 1.15E-01 |
| rs9987289 | 0.002 | 0.003 | 4.68E-01 |
| rs1508816 | -0.001 | 0.002 | 5.08E-01 |
| rs2419604 | -0.001 | 0.002 | 7.41E-01 |
| rs6982502 | 0.002 | 0.002 | 2.87E-01 |
| **Occupational position (low/intermediate/high)** | | | |
| rs9302635 | 0.036 | 0.020 | 7.71E-02 |
| rs9987289 | -0.048 | 0.027 | 7.25E-02 |
| rs1508816 | -0.019 | 0.019 | 3.22E-01 |
| rs2419604 | -0.021 | 0.017 | 2.14E-01 |
| rs6982502 | -0.001 | 0.015 | 9.28E-01 |

SNPs, single-nucleotide polymorphisms; SE, standard error

**Table S8** Association analyses between acetoacetate instrumental SNPs and potential confounders, including alcohol (ever/never), diabetes (yes/no), adiposity, i.e., waist-to-hip ratio, and occupational position (low/intermediate/high).

| **SNPs** | **Beta Coefficient** | **SE** | **P-value** |
| --- | --- | --- | --- |
| **Diabetes (yes/no)** |  |  |  |
| rs964184 | 0.004 | 0.004 | 2.89E-01 |
| rs2169387 | 0.012 | 0.005 | 1.02E-02 |
| rs1508816 | 0.003 | 0.003 | 3.54E-01 |
| **Smoking (ever/never)** |  |  |  |
| rs964184 | 0.016 | 0.011 | 1.35E-01 |
| rs2169387 | 0.020 | 0.013 | 1.06E-01 |
| rs1508816 | -0.012 | 0.009 | 1.98E-01 |
| **Alcohol consumption (heavy/other)** | |  |  |
| rs964184 | -0.035 | 0.018 | 5.08E-02 |
| rs2169387 | 0.001 | 0.021 | 9.49E-01 |
| rs1508816 | -0.016 | 0.015 | 3.01E-01 |
| **Waist-to-Hip Ratio** |  |  |  |
| rs964184 | 0.001 | 0.003 | 6.91E-01 |
| rs2169387 | 0.002 | 0.003 | 4.68E-01 |
| rs1508816 | -0.001 | 0.002 | 5.08E-01 |
| **Occupational position (low/intermediate/high)** | | |  |
| rs964184 | 0.012 | 0.022 | 5.78E-01 |
| rs2169387 | -0.048 | 0.027 | 7.25E-02 |
| rs1508816 | -0.019 | 0.019 | 3.22E-01 |

SNPs, single-nucleotide polymorphisms; SE, standard error

**Table S9** MR of BOHBUT on cognitive performance and Alzheimer's disease using 5 instrumental SNPs associated with BOHBUT (GWAS threshold: P < 5 × 10^-8^).

| **Outcomes** | **Method** | **Beta Coefficient** | **SE** | **Odds ratio** | **P-value** | **Note** |
| --- | --- | --- | --- | --- | --- | --- |
| Cognitive performance | IVW | 0.079 | 0.031 | - | 1.03E-02 |  |
|  | W-Median | 0.087 | 0.033 | - | 9.60E-03 |  |
|  | W-mode | 0.126 | 0.056 | - | 8.56E-02 |  |
|  | MR-Egger | 0.153 | 0.116 | - | 2.79E-01 | P_pleiotropy_ = 0.552 |
|  | MR-PRESSO | 0.079 | 0.031 | - | 6.22E-02 | P_global test_ = 0.277 |
| Alzheimer's disease | IVW | -0.308 | 0.142 | 0.735 | 3.06E-02 |  |
|  | W-Median | -0.319 | 0.166 | 0.727 | 5.43E-02 |  |
|  | W-mode | -0.327 | 0.220 | 0.721 | 2.12E-01 |  |
|  | MR-Egger | -0.039 | 0.504 | 0.961 | 9.43E-01 | Ppleiotropy = 0.617 |
|  | MR-PRESSO | -0.308 | 0.061 | 0.735 | 7.38E-03 | P_global test_ = 0.959 |

IVW, inverse-variance weighted; W-Midian, weighted median; W-mode, weighted mode; MR-PRESSO, Mendelian randomization pleiotropy residual sum and outlier; SE, standard error.

**Table S10** MR of BOHBUT on cognitive performance and Alzheimer's disease using 19 instrumental SNPs associated with BOHBUT (GWAS threshold: P < 1 × 10^-5^).

| **Outcomes** | **Method** | **Beta Coefficient** | **SE** | **Odds ratio** | **P-value** | **Note** |
| --- | --- | --- | --- | --- | --- | --- |
| Cognitive performance | IVW | 0.061 | 0.020 | - | 2.03E-03 |  |
|  | W-Median | 0.057 | 0.025 | - | 2.30E-02 |  |
|  | W-Mode | 0.044 | 0.047 | - | 3.67E-01 |  |
|  | MR-Egger | 0.100 | 0.055 | - | 8.66E-02 | Ppleiotropy = 0.459 |
|  | MR-PRESSO | 0.061 | 0.020 | - | 6.36E-03 | Pglobal test = 0.129 |
| Alzheimer's disease | IVW | -0.205 | 0.103 | 0.815 | 4.59E-02 |  |
|  | W-Median | -0.261 | 0.141 | 0.770 | 6.47E-02 |  |
|  | W-Mode | -0.277 | 0.178 | 0.758 | 1.41E-01 |  |
|  | MR-Egger | -0.181 | 0.325 | 0.834 | 5.87E-01 | Ppleiotropy = 0.940 |
|  | MR-PRESSO | -0.205 | 0.096 | 0.815 | 5.17E-02 | Pglobal test = 0.613 |

IVW, inverse-variance weighted; W-Midian, weighted median; W-mode, weighted mode; MR-PRESSO, Mendelian randomization pleiotropy residual sum and outlier; SE, standard error.

**Table S11** Heterogeneity and pleiotropy tests in MR of BOHBUT on cognitive performance and Alzheimer’s disease.

| **Outcomes** | **Heterogeneity test** | | **Pleiotropy test** | | **MR-PRESSO** |
| --- | --- | --- | --- | --- | --- |
|  | **Q** | **P-value (Cochran’s Q)** | **Egger intercept** | **P-value (Intercept)** | **P-value  (Global Test)** |
| **Using 5 instrumental SNPs associated with BOHBUT** |  |  |  |  |  |
| Cognitive performance | 5.636 | 2.28E-01 | -0.005 | 5.52E-01 | 2.77E-01 |
| Alzheimer's disease | 0.741 | 9.46E-01 | -0.017 | 6.17E-01 | 9.59E-01 |
| **Using 19 instrumental SNPs associated with BOHBUT** |  |  |  |  |  |
| Cognitive performance | 25.305 | 1.17E-01 | -0.002 | 4.59E-01 | 1.29E-01 |
| Alzheimer's disease | 12.329 | 5.80E-01 | -0.001 | 9.40E-01 | 6.13E-01 |

SNPs, single-nucleotide polymorphisms; MR-PRESSO, Mendelian randomization pleiotropy residual sum and outlier.

**Table S12** MR of ACACE on cognitive performance and Alzheimer's disease using 3 instrumental SNPs associated with ACACE (GWAS threshold: P < 5 × 10^-8^).

| **Outcomes** | **Method** | **Beta Coefficient** | **SE** | **Odds ratio** | **P-value** | **Note** |
| --- | --- | --- | --- | --- | --- | --- |
| Cognitive performance | IVW | 0.049 | 0.036 | - | 1.64E-01 |  |
|  | W-Median | 0.062 | 0.036 | - | 8.35E-02 |  |
|  | W-Mode | 0.076 | 0.047 | - | 2.44E-01 |  |
|  | MR-Egger | 0.101 | 0.231 | - | 7.37E-01 | Ppleiotropy = 0.857 |
|  | MR-PRESSO | NA | NA | NA | NA | Pglobal test = NA |
| Alzheimer's disease | IVW | -0.198 | 0.221 | 0.820 | 3.70E-01 |  |

IVW, inverse-variance weighted; W-Midian, weighted median; W-mode, weighted mode; MR-PRESSO, Mendelian randomization pleiotropy residual sum and outlier; SE, standard error.

**Table S13** MR of ACACE on cognitive performance and Alzheimer's disease using 14 instrumental SNPs associated with ACACE (GWAS threshold: P < 1 × 10^-5^).

| **Outcomes** | **Method** | **Beta Coefficient** | **SE** | **Odds ratio** | **P-value** | **Note** |
| --- | --- | --- | --- | --- | --- | --- |
| Cognitive performance | IVW | -0.002 | 0.026 | - | 9.41E-01 |  |
|  | W-Median | 0.007 | 0.029 | - | 8.15E-01 |  |
|  | W-mode | 0.040 | 0.039 | - | 3.26E-01 |  |
|  | MR-Egger | 0.063 | 0.072 | - | 4.00E-01 | Ppleiotropy = 0.353 |
|  | MR-PRESSO | -0.002 | 0.026 | - | 9.42E-01 | Pglobal test = 0.037 |
| Alzheimer's disease | IVW | -0.220 | 0.120 | 0.802 | 6.63E-02 |  |
|  | W-Median | -0.233 | 0.152 | 0.792 | 1.26E-01 |  |
|  | W-mode | -0.294 | 0.226 | 0.745 | 2.21E-01 |  |
|  | MR-Egger | -0.112 | 0.349 | 0.894 | 7.55E-01 | Ppleiotropy = 0.750 |
|  | MR-PRESSO | -0.220 | 0.055 | 0.802 | 2.46E-03 | Pglobal test = 0.995 |

IVW, inverse-variance weighted; W-Midian, weighted median; W-mode, weighted mode; MR-PRESSO, Mendelian randomization pleiotropy residual sum and outlier; SE, standard error.

**Table S14** Heterogeneity and pleiotropy tests in MR of ACACE on cognitive performance and Alzheimer’s disease.

| **Outcomes** | **Heterogeneity test** | | **Pleiotropy test** | | **MR-PRESSO** |
| --- | --- | --- | --- | --- | --- |
|  | **Q** | **P-value (Cochran’s Q)** | **Egger intercept** | **P-value (Intercept)** | **P-value  (Global Test)** |
| **Using 3 instrumental SNPs associated with ACACE** |  |  |  |  |  |
| Cognitive performance | 3.068 | 2.16E-01 | -0.004 | 8.57E-01 | NA |
| Alzheimer's disease | 0.308 | 5.79E-01 | NA | NA | NA |
| **Using 14 instrumental SNPs associated with ACACE** |  |  |  |  |  |
| Cognitive performance | 22.269 | 3.46E-02 | -0.004 | 3.53E-01 | 3.70E-02 |
| Alzheimer's disease | 2.092 | 9.96E-01 | -0.007 | 7.50E-01 | 9.95E-01 |

SNPs, single-nucleotide polymorphisms; MR-PRESSO, Mendelian randomization pleiotropy residual sum and outlier

**Table S15** MR of BOHBUT on cognitive performance and Alzheimer's disease using 4 instrumental SNPs (rs6982503 was removed) associated with BOHBUT (GWAS threshold: P < 5 × 10^-8^).

| **Outcomes** | **Method** | **Beta Coefficient** | **SE** | **Odds ratio** | **P-value** | **Note** |
| --- | --- | --- | --- | --- | --- | --- |
| Cognitive performance | IVW | 0.093 | 0.032 | - | 3.43E-03 |  |
|  | W-Median | 0.115 | 0.035 | - | 9.99E-04 |  |
|  | W-mode | 0.129 | 0.051 | - | 8.65E-02 |  |
|  | MR-Egger | 0.081 | 0.160 | - | 6.61E-01 | P_pleiotropy_ = 0.949 |
|  | MR-PRESSO | 0.093 | 0.032 | - | 6.12E-02 | P_global test_ = 0.348 |
| Alzheimer's disease | IVW | -0.274 | 0.153 | 0.760 | 7.22E-02 |  |
|  | W-Median | -0.302 | 0.172 | 0.739 | 7.91E-02 |  |
|  | W-mode | -0.331 | 0.222 | 0.718 | 2.32E-01 |  |
|  | MR-Egger | -0.155 | 0.628 | 0.856 | 8.28E-01 | P_pleiotropy_ = 0.863 |
|  | MR-PRESSO | -0.274 | 0.054 | 0.760 | 1.48E-02 | P_global test_ = 0.955 |

IVW, inverse-variance weighted; W-Midian, weighted median; W-mode, weighted mode; MR-PRESSO, Mendelian randomization pleiotropy residual sum and outlier; SE, standard error.

**Table S16** *cis-*SNPs in *OXCT1* (GWAS threshold: P < 1 × 10^-5^) using clumping window -/+ 10 000 kb and LD-r^2^ < 0.4.

| **SNPs** | **Beta Coefficient** | **SE** | **P-value  (Discovery Stage)** | **Effect Allele** | **Effect Allele (Frequency)** |
| --- | --- | --- | --- | --- | --- |
| rs1508816 | 0.052 | 0.008 | 5.04E-11 | T | 0.745 |
| rs57944767 | -0.077 | 0.017 | 4.11E-06 | A | 0.048 |
| rs79498594 | -0.057 | 0.013 | 6.50E-06 | T | 0.101 |

SNPs, single-nucleotide polymorphisms; SE, standard error; Sample size = 45,031; F-statistic > 15.

**Table S17** *cis*-MR for a causal estimate of *OXCT1* locus on cognitive performance and Alzheimer's disease.

| **Outcomes** | **Method** | **Beta Coefficient** | **SE** | **P-value** | **Note** |
| --- | --- | --- | --- | --- | --- |
| Cognitive performance | IVW | 0.038 | 0.054 | 0.312 |  |
|  | MR-Egger | -0.013 | 0.326 | 1.138 | Ppleiotropy = 0.869 |
| Alzheimer's disease | IVW | -0.331 | 0.312 | 0.29 |  |
|  | MR-Egger | -0.199 | 1.138 | 0.861 | Ppleiotropy = 0.904 |

IVW, inverse-variance weighted, SE, standard error.

## ****References****

1. Machado-Fragua MD, Landre B, Chen M, Fayosse A, Dugravot A, Kivimaki M, et al. Circulating serum metabolites as predictors of dementia: a machine learning approach in a 21-year follow-up of the Whitehall II cohort study. BMC Med. 2022;20(1):334.

2. Soininen P, Kangas AJ, Wurtz P, Suna T, Ala-Korpela M. Quantitative serum nuclear magnetic resonance metabolomics in cardiovascular epidemiology and genetics. Circ Cardiovasc Genet. 2015;8(1):192-206.

3. Shah T, Engmann J, Dale C, Shah S, White J, Giambartolomei C, et al. Population genomics of cardiometabolic traits: design of the University College London-London School of Hygiene and Tropical Medicine-Edinburgh-Bristol (UCLEB) Consortium. PLoS One. 2013;8(8):e71345.

4. Kettunen J, Demirkan A, Wurtz P, Draisma HH, Haller T, Rawal R, et al. Genome-wide study for circulating metabolites identifies 62 loci and reveals novel systemic effects of LPA. Nat Commun. 2016;7:11122.

5. Yurista SR, Chong CR, Badimon JJ, Kelly DP, de Boer RA, Westenbrink BD. Therapeutic Potential of Ketone Bodies for Patients With Cardiovascular Disease: JACC State-of-the-Art Review. J Am Coll Cardiol. 2021;77(13):1660-9.

6. Hu E, Du H, Zhu X, Wang L, Shang S, Wu X, et al. Beta-hydroxybutyrate Promotes the Expression of BDNF in Hippocampal Neurons under Adequate Glucose Supply. Neuroscience. 2018;386:315-25.

7. Chung JY, Kim OY, Song J. Role of ketone bodies in diabetes-induced dementia: sirtuins, insulin resistance, synaptic plasticity, mitochondrial dysfunction, and neurotransmitter. Nutr Rev. 2022;80(4):774-85.
